# *Taenia solium* Cysticercosis and Taeniosis Reporting in the Current Medical and Veterinary Diseases Reporting Systems in Tanzania: A Cross-Sectional Study

**DOI:** 10.1155/2024/5592872

**Published:** 2024-02-17

**Authors:** Fredy Mlowe, James Mlangwa, Ernatus Mkupasi, Andrea S. Winkler, Antony D. Nyerere, Ayubu Churi, Helena Ngowi, Esron Karimuribo

**Affiliations:** ^1^Department of Veterinary Medicine and Public Health, College of Veterinary Medicine and Biomedical Sciences, Sokoine University of Agriculture, Morogoro, Tanzania; ^2^Center for Global Health, Department of Neurology, Technical University of Munich, Munich, Germany; ^3^Center for Global Health, Institute of Health and Society, University of Oslo, Oslo, Norway; ^4^Department of Informatics and Information Technology, Sokoine University of Agriculture, Morogoro, Tanzania

## Abstract

*Taenia solium* cysticercosis and taeniosis (TSCT) are two forms of a zoonotic disease caused by *T. solium* tapeworm. Towards promotion of a One Health approach to the control of TSCT, we assessed TSCT reporting in the medical and veterinay sectors in Tanzania. We conducted a cross-sectional study between January and April 2020 in Babati and Mbulu districts (northern Tanzania), Kongwa district (central Tanzania), Mbinga and Nyasa districts (southern Tanzania), and the Zonal Veterinary Centres in Iringa (southern Tanzania) and Arusha (northern Tanzania) regions. A questionnaire was administered to 154 officers in charge (OsIC) of primary healthcare facilities (PHFs) and 110 meat inspectors (MIs) to collect quantitative data. Key informant interviews (KIIs) were conducted to 16 medical and 17 veterinary officers from level one healthcare facilities and district livestock offices, respectively, to their respective ministries. OsIC admitted absence of specific reporting (100%, *n* = 154) of *T. solium* taeniosis and neurocysticercosis (NCC) in the medical diseases reporting system (MDRS) despite the presence of optimum facilitation in terms of report preparation and submission (92.2%, *n* = 154) with 83.8% (*n* = 154) timely report submission rate. The veterinary diseases reporting system (VDRS) accommodated porcine cysticercosis (PCC) reporting. Nevertheless, approximately 77.3% (*n* = 110) of the MIs admitted inadequate facilitation in VDRS that hindered efficient reporting of PCC among other diseases. In addition, all MIs admitted that disease reports submitted were incomplete, submitted late (73.3%, *n* = 110), or not submitted at all (88.8%, *n* = 110). Similarly, KIIs results revealed suboptimal facilitation and reporting efficiency in VDRS than it was with the MDRS. It is concluded that the MDRS did not provide for specific reporting of taeniosis and NCC. Inadequate facilitation of the general VDRS hindered efficient PCC reporting despite its provision for PCC reporting. A One Health approach in strengthening the medical and veterinary diseases reporting systems for efficient TSCT reporting is recommended.

## 1. Introduction

*Taenia solium* tapeworm is a parasite of public health importance that infects human and pigs. The tapeworm causes *T. solium* cysticercosis/taeniosis (TSCT) and neurocysticercosis (NCC). The parasite is transmitted between humans who are the definitive hosts and pigs which are the intermediate hosts and between humans themselves [1]. Risky practices that sustain the life cycle of the parasite include unhygienic practices in food preparation, poor sanitation, and undercooking of pork. Furthermore, when free ranging pigs in areas with inadequate latrine use access human feces or eat and drink on feeds and water contaminated with *T. solium* eggs, parasite transmission between human and pigs is sustained [2].

Several studies have established TSCT burden in different areas in Tanzania. Using enzyme-linked immonosorbent assay (ELISA) and Kato-katz, a taeniosis prevalence of 5.2% and 2.3%, respectively, has been established [3]. Antigen (Ag) ELISA has been able to establish 16.3% burden of human cysticercosis in Mbulu, out of which 19% had lesions implicated with NCC under CT scan [4] while 16.7% prevalence has been established in Mbozi [3].

*T. solium* taeniosis, which is the presence of adult form of the tapeworm in the small intestines of a human, is characterised by abdominal discomforts which are nonspecific [5]. NCC is the most clinically important and life-threatening form of the tapeworm whose main clinical manifestations are late acquired epilepsy and chronic headache [5]. Up to 32% of all late onset epileptic cases have been attributable to human NCC in endemic areas [5]. Similar clinical manifestations of NCC have been shown in pigs [6].

Successful control and eradication of the disease in both humans and pigs require adequate and reliable knowledge on the burden and distribution of the parasite in both pigs and humans [6]. On the other hand, the reliable knowledge on the disease burden and distribution relies on efficient and coordinated reporting of the diagnosed cases of the disease in both medical and veterinary sectors [7]. Thus, monitoring and reporting of PCC, human NCC, and taeniosis are among the important elements for the effective control of *T. solium* taeniosis/cysticercosis [8]. However, TSCT and NCC are among the neglected diseases and rarely reported in many countries [9]. This fact is reflected in the deficiency of data about the disease in most European [10] and African [11] countries. As a matter of fact, most of the epidemiological and burden data about TSCT disease in most of the endemic African countries such as Zambia are much more based on research findings [12–14] than on routine diseases surveillance programs. Furthermore, what is known about TSCT prevalence, epidemiology, health, and economic burdens in Mozambique is the result of years of intensive research on the disease in the country [15–17].

Similarly, much of what is known about *T. solium* taeniosis, NCC, and PCC national status in both medical and veterinary sectors in Tanzania is mainly through report findings from various research' programs conducted in various areas within the country [18]. The Ministry of Health has designed a Health Management Information System (HMIS) which among other functions, it provides for reporting of all health events identified within the healthcare provision facilities [19]. On the other hand, the Ministry of Livestock and Fisheries Development (MLFD) has an Epidemiology Unit (EU) under the Directorate of Veterinary Services (DVS) whose functions include livestock diseases' surveillance and monitoring among others. The unit gathers countrywide veterinary diseases status from multiple sources, including the livestock field extension officers through the District Veterinary Officers who are obliged to report diseases events by the animal diseases act of 2003 [20]. Nevertheless, George et al. [21] reported inefficiency of the current VDRS, which is contributed by the lack of funding, supporting systems, and communication challenges, among others.

The aim of this study was to assess how *T. solium* taeniosis, NCC, and PCC diseases are accommodated in the routine national diseases surveillance and reporting systems in both medical and veterinary diseases surveillance and reporting systems in Tanzania.

## 2. Materials and Methods

### 2.1. Description of the Study Areas

The study was conducted in five purposively selected regions of Tanzania, namely, Ruvuma, Dodoma, Manyara, Arusha, and Iringa. Details about the study sites are as described by Mlowe et al. [22].

### 2.2. Study Design

A cross-sectional study was conducted from January to April 2020, involving quantitative and qualitative methods of data collection. Interviews were conducted using a structured questionnaire to collect quantitative data from Officers in Charge (OsIC) of Primary Healthcare Facilities (PHFs) and Meat Inspectors (MIs) while one-to-one in-depth interviews were conducted with key informants to collect qualitative data.

### 2.3. Sample Size and Participants Selection

The number of respondents for quantitative data was calculated using the following formula: *n* = *N*/(1 + *N*(*e*)^2^), where *n* = study sample size, *N* = study population size, and *e* = level of precision. The number of OsIC of PHFs and MIs included in the study from each district was determined based on the probability proportional to the size sampling approach. The number of livestock and agricultural field officers doing meat inspection in wards and villages in the veterinary sector was recruited into the district sample size based on the proportion each of the two strata contributed to the total number of extension officers in each district. Both MIs and OsIC of PHFs were randomly selected from the list of MIs and PHFs in the district provided by the District Veterinarian or Livestock Officer and District Medical Officer, respectively.

Respondents for qualitative data from the medical and veterinary sectors were purposively selected from level I healthcare provision facilities (private and public hospitals) and District Livestock and Fisheries Development Department (DLFDD), respectively, to their respective ministries. Their number was determined based on the saturation point reached. They were interviewed using one-to-one key informant interview based on the criteria that they are experts in the field to ensure that they provide expertise opinion regarding a research topic.

### 2.4. Data Collection

Quantitative data were collected from MIs and medical health respondents who were OsIC of PHFs. Qualitative data were collected from both medical and veterinary officers working from level I healthcare provision facilities (public or private level I hospital) and the DLFDD, respectively, to their respective ministries. The medical respondents targeted for qualitative data were the medical doctors (MDs). Respondents targeted from the veterinary sector were the districts' Veterinary Officers (DVOs) or District Livestock and Fisheries Officer (DLFO) in the absence of a DVO at the district level. Veterinary doctor or livestock officer (in the absence of veterinary doctor) at any higher level beyond the district level was the targeted respondent. General questions reflecting the ideal diseases reporting (timeliness, completeness, and report quality) [23] were asked. Specific questions reflecting TSCT and NCC accommodation within the existing medical diseases surveillance and reporting systems or a stand-alone reporting channel were inquired. In addition, the presence of epilepsy reporting, which is the common clinical presentation of NCC was asked. This was in an attempt to figure out the potential of the current diseases reporting system to capture at least the potential signs for the presence of NCC in endemic areas. Quantitative data were collected using a tablet and a smart phone in which structured questionnaires were digitalized in both using Afyadata software [24]. In addition, structured questionnaires for both (OsIC of PHFs and MI) were pretested to 10 respondents each and corrected accordingly before they were actually used for data collection. Qualitative data were collected using audio recorder following a verbal consent given by a respondent. Mlowe et al. [22] further described details of both qualitative and quantitative data collection.

### 2.5. Ethical Approval

Ethical approval and participants' consent to participate in the study were as described by Mlowe et al. [22].

### 2.6. Data Analysis

#### 2.6.1. Quantitative Data Analysis

Quantitative data were analysed in SPSS for frequencies and proportions following retreieving of the data from the Afyadata softwere saver into Excel spreadsheet. Regarding taeniosis, NCC/epilepsy, and PCC reporting, the following variables were analyzed: the presence of reporting format and specificity in reporting of taeniosis, NCC/epilepsy, and PCC. In addition, the availability of reporting facilitation to OsIC and MIs, timeliness in report submission, completeness in reporting, and the means of sending and getting the reporting feedback were analyzed .

#### 2.6.2. Qualitative Data Analysis

Qualitative data (recorderd audios) were transcribed and analysed as described by Mlowe et al. [22].

## 3. Results

### 3.1. Quantitative Results

A total of 264 respondents, out of which 154 (58.3%) were OsIC of PHFs, were interviewed. Of 154 OsIC interviewed, 133 (86.4%) were from dispensaries and 21 (13.6%) were from healthcare centres (primary healthcare facilities). In addition, 191 (72.3%) of the respondents were males.  Table 1 describes demographic characteristics of the interviewed respondents.

#### 3.1.1. Assessment of *Taenia solium* Taeniosis and Epilepsy Reporting

All PHFs had a register book called the health management information system (HMIS) register book for registering all cases attended at a particular health facility with all necessary details about the disease and patient. However, 100% of the respondents said that there was no specific reporting format specifically designed to report taeniosis or epilepsy. In addition, 92.2% of the respondents admitted that they were facilitated in many aspects in report preparation and submission and 83.8% were capable of submitting the reports to the respective authority on time. The remaining 16.2% of the respondents who could not submit reports on time had various reasons for the delay, with 51.3% of the respondents admitting that they were overwhelmed by health services provision duties (Table 2).

Regarding the means of sending the reports, 43.8% of the respondents said they normally sent the reports physically while the rest were sending via other methods including phone text message (22.3%), someone they knew or trusted (14.8%), and email or through an electronic report submission system (18.8%). OsIC of PHFs had various ways of getting reporting feedback as summarized in Table 3.

#### 3.1.2. Porcine Cysticercosis Reporting

The study found that most MIs had no specific register book to record daily pig inspection findings. Each MI had his or her own means of recording meat inspection findings, whereby 54% of the MIs used notebooks to record their findings.  Table 4 summarizes the means by which MIs used to record meat inspection findings.

Out of the 110 respondents interviewed, 91.8% admitted absence of specific reporting forms for PCC. In addition, 77.3% of the respondents admitted absence of reporting facilitation, with 18.2% of those facilitated being facilitated mainly by the DLFO, of which 70% were facilitated mostly in terms of stationeries (Table 5).

Out of the 110 MIs interviewed, 44.5% used the agriculture routine data system (ARDS) forms to report PCC and other livestock diseases, 26.4% used locally designed abattoir reports and 8.2% used abattoir diseases surveillance forms. The remaining 20.9% prepared reports using various other methods including both ARDS and diseases surveillance forms, any forms at their convenience, and some admitted that they were not sending abattoir or any disease report. Furthermore, 87.3% of the respondents said that they used to send the reports direct to the District Livestock and Fisheries Office (DLFO). Out of the remaining respondents, 12.7% mentioned several other routes through which the reports were channeled before reaching the DLFO office. In addition, 49.4% of the MIs said to have submitted the reports physically and 31.9% used the people they knew or trusted to send the reports to DLFO office. The remaining respondents sent the reports using either phone text message, WhatsApp, phone calls, or physically sent only when they had other personal agenda at the district headquarters.

Regarding reporting feedback, 37.9% of the respondents said they were sure that the reports had reached the respective office because they sent them in person and, therefore, there was no need to find other ways to confirm, while 25% of those who sent through other means had to call to the office to be sure that the reports had reached the respective office, among other means.

Respondents who could not submit reports on time had various reasons for reporting delay, with 41.8% of the respondents being too much overwhelmed by field activities to get time to write the report, 14.5% saying they were not facilitated by any means, and 9.1% saying that bad weather and infrastructures during rainy season delayed them in sending the report.

### 3.2. Qualitative Results

A total of 33 people were interviewed.  Table 6 summarises the demographic characteristics of the respondents. Three of the five districts had two councils, district council and town council, each with a district and town medical and veterinary doctor. This accounted for a total number of eight districts and six town councils' medical and veterinary doctors interviewed.

#### 3.2.1. Taenia *solium* Taeniosis Reporting

A total of 16 health personnel (eight from district/town council hospitals, three from regional hospitals, and one from Ministry of Health, Community Development, Gender, Elderly, and Children (MoHCDEC) were interviewed to capture their perceptions on the availability of *T. solium* taeniosis reporting. Sixteen respondents admitted that taeniosis disease was not specifically reported in isolation from other worm infestations. Rather, it was reported under a group of “intestinal worms” in general (“… *If you go into the register book you may specify either the doctor has diagnosed which type of worms but the challenge comes in the tallying book, where you have to write worms, you only have to write intestinal worms*.” Female, HMIS Regional focal person). In addition, nine respondents admitted the absence of reporting format specifically designed to report taeniosis only (“*it has been grouped in intestinal worms, it doesn't have its separate reporting format*.” Female Ag. District Medical Doctor).

#### 3.2.2. Epilepsy Reporting

A total of 14 health respondents (eight from district/town council hospitals, five from regional hospitals, and one from MoHCDEC) were interviewed to capture their perceptions on the availability of epilepsy routine reporting. Seven out of eight respondents admitted that epilepsy was not reported to the level of identifying a specific cause (“*Yes, it is there, if we get time we shall access the system and see although they don't appear as they are, you will find it in general as epilepsy*,” Male, District Medical Officer). In addition, all 7 respondents admitted that there was no reporting format specifically designed to report epilepsy cases only (“*Epilepsy is there with other diseases, it appears as epilepsy in general*,” Male, District Medical Officer).

#### 3.2.3. Neurocysticercosis Reporting

A total of 14 health respondents (eight from district/town councils and six from regional hospitals) were interviewed to capture their perceptions on the NCC routine reporting. Four out of five respondents admitted that there was no specific reporting for NCC (“*Aaah, that cysticercosis, first of all it is among the neglected diseases, why am I saying so? That disease is not reported anywhere in the disease reporting systems of the Ministry of Health, we are talking about the health sector*…,” Male, Epidemiologist at MoHCDEC).

#### 3.2.4. General Disease Reporting in the Medical Sector

A total of 14 health officials (eight from district/town councils and six from regional hospitals) were interviewed to capture their perceptions on general disease reporting routine in the medical sector; particularly, report completeness, reporting facilitation, report quality check, and timeliness. Ten out of 12 respondents admitted that there was reporting facilitation which included among others, provision of reporting tools, and bus fare for submitting the reports (“*Yeah, they have it, they have it because the government is sending money to all health providing facilities direct and they are the signatories themselves, so they have it in the budget, bus fare …*,” Male, District Medical Officer), while seven out of 13 respondents admitted that reports were timely submitted (“*Yeah, in our case, they submit on time,* …,” Male, Town Council Medical Doctor). In addition, nine out of 15 respondents admitted that almost all health facilities submitted the reports (“*almost 100% of health facilities submit the reports*,” Regional Medical Doctor, Male), and six out of 15 respondents admitted that the reports were checked for quality before submission.

#### 3.2.5. Porcine Cysticercosis Reporting

Fifteen respondents (eight from district/town council livestock and fisheries offices, three from regional administrative secretariat, three from ZVC, and one from the Ministry of Livestock and Fisheries) were interviewed to capture their perceptions on PCC reporting along the veterinary sector disease reporting system. Five out of six quotations admitted that PCC reporting was disease specific, meaning that the reporting allowed the reporter to name the disease specifically as he or she has diagnosed it (“*This format is self-sufficient, because it is the matter of the extension officers to identify the cyst and report it, but if the extension officer has got no ability to identify, then that is a challenges*,” Male, Regional Veterinary Doctor) while one of the respondents admitted that there was no specific PCC reporting, meaning that the reporter would just write cyst without telling which kind of cyst exactly is he or she referring to. In addition, there were 10 respondents who admitted that there was no format for PCC reporting, meaning that PCC had no independent format for disease reporting; instead, it was reported together with other diseases (“*I have not seen it in this district, let me say this,…we don't have specific format for porcine cysticercosis reporting,* …,” Male, District Veterinary Doctor).

Five respondents admitted that there was quality check of disease reports before submission to the next reporting level (“*We say one of our responsibilities here is to collect and disseminate the reports,.… You may need to call to verify...*,” Male, Veterinary Doctor, ZVC).

#### 3.2.6. General Veterinary Diseases Reporting

Fifteen respondents (eight from district/town council livestock and fisheries offices, three from regional administrative secretariat, three from ZVC, and one from the Ministry of Livestock and Fisheries) were interviewed to capture their perceptions regarding veterinary diseases reporting in general. A total of 11 out of 15 respondents admitted that reports were always submitted after submission deadline (“*Aaah, not easy,.…, Wednesday is the day for report submission … you can find yourself forced to send the report on Thursday sometimes up to Sunday*,” Female, Acting District Livestock and Fisheries Officer), while eight out of nine admitted that sometimes reports were not submitted (“*….there in Hanang, Mbulu and the like…we don't get the inspection reports from those areas*,” Male, Veterinary Doctor, in Charge, ZVC). Fourteen out of 15 respondents admitted that there was no any facilitation in disease report preparation and submission (“*Aaah, there is no specific facilitation to people working in the field for report submission,…*,” Male, Veterinary Doctor, In charge, ZVC), while five out of six respondents admitted that there was no consistence in disease reporting among meat inspectors (*…. it's until you call them, begging them to send the report. It's very challenging honestly'*, Male, Regional Veterinary Doctor). Furthermore, all two respondents regarding completeness of the reports admitted that there was no any completeness in disease reporting (“*You see, or sometimes the report are submitted in different qualities, no uniformities in report quality*,” Male, Veterinary Doctor, In charge ZVC).

## 4. Discussion

### 4.1. Overview

The study findings have highlighted the challenges to be addressed to enable efficient TSCT disease reporting. The findings have realized the need and importance of the One Health approach to ensure that sufficient data on the disease burden and epidemiology are collected countrywide to create the basis for TSCT control and elimination strategies. In addition, the findings reflect the really global (and specifically sub-Saharan Africa) challenges in infectious (including neglected tropical) diseases surveillance and reporting [11, 25, 26]. This fact calls for furthering research on the best ways sub-Saharan state governments can deliberately develop an effective TSCT disease surveillance and reporting system that can effectively and specifically capture TSCT disease burden to suffice an effective disease control and/or elimination policy formulation within and across endemic countries.

Overall, the study findings show that *T. solium* taeniosis, NCC/epilepsy, and PCC reporting faces many challenges to enable collection of sufficient epidemiologically important data about the disease. The data are important in influencing policy and decision makers to strategize towards control and possible eradication of the disease in the country in both intermediate and definitive hosts. The challenges of reporting each form of the TSCT desease is specifically discussed below.

### 4.2. *T. solium* Taeniosis and Epilepsy/Neurocysticosis Reporting

The national MDRS is well structured, coordinated, and optimally financed to support smooth health surveillance data reporting from the PHFs to the national level. Nevertheless, *T. solium* taeniosis had no place to be reported within the surveillance and reporting systems and neither was *T. solium* NCC/epilepsy. While it was impossible to find the term “NCC” in the reporting forms, epilepsy which is the main clinical presentation of NCC, was reported as “epilepsy” and as such, the one whom the report was intended for cannot tell the exact cause of epilepsy even when a physician in a well-equipped hospital with advanced diagnostic facilities could have diagnosed the exact cause of epilepsy. This poses difficultness in estimating the magnitude of any primary cause of the disease, be it *T. solium* NCC or else, against which to strategize towards control or elimination.

The study also found that *T. solium* taeniosis and the NCC national status could not be accurately established. This was contributed by the fact that the disease does not get reported as a standalone reportable disease in the MDRS. The disease was considered less important and one of the neglected diseases in the country as it was captured from one of the respondents of one-to-one in-depth key informant interview.

*T. solium* NCC has contributed about 7.4% (*p* < 0.0000), 3.3% (*p* < 0.0001), and 1.1% (*p* < 0.04) prevalence of epileptic cases in Mbozi, Mbulu, and Hai districts, respectively [18]. These data are from research findings from specific studies done in the respective districts and not from the routine MDRS. This fact further supports our findings that the routine MDRS currently in place is not supportive enough to accurately quantify taeniosis and NCC infection burden. A review to the current MDRS such that it provides for TSCT reporting could ensure reliable and accurate data estimation on the disease burden that would form the basis of informing the policy and subsequently strategise on the cost effective TSCT control options.

Absence of specificity in helminthosis/taeniosis reporting as was found out by this study further intensifies the difficultness in quantifying taenioisis cases at any level of healthcare provision facility. Likewise, systematic review studies aimed at establishing epidemiology of *T. solium* taeniosis and cysticercosis in Europe reported absence of species specificity in reporting identified taeniosis cases among the challenges hindering the TSCT magnitude estimate [10]. However, different from our study findings, initiatives in reporting taeniosis and cysticercosis cases in European countries [10] stand higher chance to improve the accuracy of the disease magnitude estimate than it is with our country. Nevertheless, similar to our study, both studies show that taeniosis and human cysticercosis are still neglected and marginalized diseases in the existing diseases surveillance and reporting systems, resulting in under-reporting and obscured data on the disease prevalence. As a result, the global disease burden is likely to be underestimated as well.

### 4.3. Porcine Cysticercosis Reporting

The livestock disease surveillance and reporting systems allowed livestock diseases to be reported the way they were diagnosed by field veterinarians. This provided equal chance for *T. solium* PCC reporting as long as the field extension officer was knowledgeable and capable of diagnosing the disease. However, the absence of uniformity and consistence in report content and quality as explained by the lack of general disease reporting facilitation (Table 5) hinders the quality and timely availability of the reports. It is generally realized from this study that *T. solium* PCC adequate reporting relies on the efficiency of the general VDRS. This is because there is no specific reporting or surveillance plan for the disease currently in place. Thus, inefficiency of the general VDRS reflects inefficient reporting of *T. solium* PCC as well. In addition, the general VDRS is not adequately supported to enable efficient reporting of veterinary diseases. This is evidenced by inadequate material and financial support to the whole surveillance and reporting system, including inadequate facilitation to extension officers in the field for data gathering and reporting (Table 5).

Inadequate MIs and absence of facilitation to livestock extension officers working in the field and along the reporting chain was found to be one of the limiting challenges for effective PCC and other livestock diseases reporting in general. Likewise, a study in Uganda reported inadequate number of staff and poor motivation and communication along the surveillance chain among the contributing factors for inadequate performance of the diseases reporting [26], indicating common diseases surveillance and reporting challenges in sub-Saharan Africa, which may need common approaches to strengthening them.

While fewer reports for PCC in most of the European countries was partly contributed by the absence or fewer PCC cases diagnosed as a result of an improved management and confinement of pigs with improved sanitary conditions [10], fewer cases in our study areas was contributed by under-reporting of the disease, contributed by inadequate number of MIs to diagnose and report *T. solium* PCC positive cases, reluctance of some field officers to report cases, and inadequate facilitation of the livestock field officers, reporting staff and the VDRS in general.

### 4.4. *T. solium* Taeniosis, Epilepsy/Neurocysticercosis, and Porcine Cysticercosis Reporting Compared

The MDRS is by far much more efficient in some aspects of ideal diseases surveillance and reporting compared to the VDRS. This is probably due to the fact that the MDRS is adequately supported in terms of resources and infrastructures than the VDRS as was found out by this study. Therefore, it is evident that *T. solium* taeniosis and NCC could be equally and efficiently reported by the healthcare provision facilities if it were given a priority and included among the reportable medical diseases. On contrary, despite the fact that most animal diseases reporting forms provided for *T. solium* PCC reporting, the general VDRS is not adequately facilitated to support efficient reporting of the *T. solium* PCC disease, among others.

The One Health approach is currently advocated for successful control and eradication of *T. solium* taeniosis and NCC [27]. However, this study found unequal level, capacity, and priorities of the existing diseases surveillance and reporting systems within and between medical and veterinary sectors towards *T. solium* taeniosis, NCC, and PCC reporting. This challenge has a potential of decelerating the efforts towards the One Health approach in controlling the disease within the country. In addition, while *T. solium* taeniosis reporting in the medical sector was limited by the lack of specific disease reporting in the routine MDRS, PCC reporting was limited by inadequate general veterinary diseases reporting support and facilitation in most of the important aspects of veterinary diseases surveillance and reporting along the reporting chain, among other factors. This fact has a potential of creating limitation of implementing the One Health approach in combating *T. solium* NCC with strategies towards controlling *T. sollium* taeniosis and PCC as the easiest measurable variables than targeting NCC itself [8]. In addition, together with other findings revealed by this study, it calls for further research on tools and innovations on how to leverage One Health and global efforts against neglected tropical diseases to streamline the available diseases surveillance and reporting systems in Tanzania and other endemic countries of sub-Saharan Africa to accommodate and improve specific reporting of TSCT, within and across the countries.

## 5. Conclusion

Routine diseases surveillance and reporting systems in both medical and veterinary sectors in Tanzania do not support effective *T. solium* taeniosis, NCC, and PCC reporting. Inadequate support in the VDRS was reflected in *T. solium* PCC under-reporting, whereas difference in priorities for surveillance and reporting of human diseases was reflected by the absence of reporting of *T. solium* taeniosis and NCC despite the optimum facilitation of the MDRS. With the current emphasis on the One Health approach in addressing *T. solium* infections, diseases surveillance systems need to be reviewed and updated to provide for routine collection of sufficient epidemiological data for successful disease control and elimination programs. In addition, considering the possible TSCT common reporting challenges among sub-Saharan countries such as Zambia and Mozambique where the disease is endemic, introducing a harmonized TSCT surveillance and reporting network which works in a One Health manner would contribute into fast pushing of a One Health agenda specific for this disease among member states for effective control of the disease in both human and pigs in the endemic countries.

## Figures and Tables

**Table 1:** Demographic characteristics of officer in charges of primary healthcare facilities and meat inspectors.

Variable	Number and percentage (%) of respondents	
	OsIC	MIs
*Region*		
Manyara	53 (34.4)	38 (34.5)
Dodoma	36 (23.4)	27 (24.5)
Ruvuma	65 (42.2)	45 (40.9)

*District*		
Babati	30 (19.5)	22 (20.0)
Mbulu	23 (14.9)	16 (14.5)
Kongwa	36 (23.4)	27 (24.5)
Mbinga	44 (28.6)	28 (25.5)
Nyasa	21 (13.6)	17 (15.5)

*Type of primary health facility*		
Dispensary	133 (86.4)	NA
Health centres	21 (13.6)	NA

*Health facility ownership*		NA
Private	36 (23.4)	NA
Public	118 (76.6)	NA

*Clinical medical health against other profession*		
Officers in charge with clinical medical health background	119 (97.3)	NA
Officers in charge with nonclinical medical health background	35 (22.7)	NA

*Animal health profession against other professions*		
Animal health	NA	43 (39.1)
Other professions	NA	67 (60.9)

**Table 2:** Causes of delay in report submission by officers in charge of healthcare provision facilities.

Variable	No. of responses (*n* = 37)	Percentage (%)
Overwhelmed by health services provision duties	19	51.3
Not facilitated by any means	1	02.7
Do not get anyone to help sending the report	1	02.7
Office headquarter very far away to send report on time	6	16.2
Poor infrastructure and bad weather during rain seasons	4	10.8
Inadequate human resource and sickness	2	05.4
Health facility owner delay on checking on WhatsApp sent reports	1	02.7
Public holidays falling on sending deadline	1	02.7
Sometimes, lack of bus fare to send someone to send the reports	1	02.7
Poor network coverage delays online submission of reports	1	02.7

**Table 3:** Means by which officers in charge of primary healthcare facilities were getting reporting feedback.

Variable	No. of responses (*n* = 283)	Percentage (%)
I usually call to ask	55	19.4
They call to acknowledge receipt of the report	42	14.8
They write a text message	42	14.8
They write an email to acknowledge receipt of the report	04	01.4
No need of reporting feedback because we submit the report in person	124	43.8
We send in person and sign and if sent electronically, we get an electronic feedback	14	04.9
I do not call but the call if they have not got the report	02	00.7

**Table 4:** Means by which meat inspectors were recording pork inspection findings.

Variable	No. of responses (*n* = 110)	Percentage (%)
Village record book	06	05.5
Meat inspection record book	27	24.5
Note book	60	54.5
Exercise book	10	09.1
Portable computer	01	00.9
Meat inspection file	01	00.9
Peace of paper	04	03.6
Do not record	01	00.9

**Table 5:** Porcine cysticercosis reporting by meat inspectors.

Variable	Number of responses (*n*)	Percentage
*Presence of reporting facilitation*		
Not applicable (excluded from previous responses)	2	1.8
Yes	23	20.9
No	85	77.3

*Timely report submission*		
Yes	55	50.0
No	10	36.4
Not applicable (excluded from previous responses)	15	13.6

*Presence of porcine cysticercosis-specific reporting format*		
Yes	9	8.2
No	101	91.8

*Sources of facilitation*		
Not facilitated	87	79.1
DLFO	20	18.2
DLFO and NGOs	1	0.9
Ministry of livestock	2	1.8

*Aspects of reporting facilitation*		
Given stationeries	21	70.0
Air time bundles	1	3.3
Given motorcycle	5	16.7
Fuel	2	6.7
Given portable computer	1	3.3

**Table 6:** Demographic characteristics of respondents of a qualitative data to assess *T. solium* taeniosis, cysticercosis, and neurocysticercosis reporting.

Factor	Number of respondents (*n*)	Percentage (%)
*Sex*		
Male	28	88.8
Female	5	11.2

*Occupation*		
Medical doctor	15	46.8
Nurse	1	03.1
Veterinary doctor	9	28.1
Animal scientist	5	15.6
Veterinary paraprofessional	2	06.3

*Work station*		
District hospital	8	24.2
Regional hospital	6	18.2
Missionary referral hospital	1	03.0
Zonal Veterinary Investigation Centre (ZVC)	3	09.1
Tanzania Veterinary Laboratory Agency (TVLA)	2	06.1
District Livestock and Fisheries Office	8	24.2
Regional Administrative Secretariat	4	12.1
Ministry of Livestock and Fisheries Development	1	03.0
Ministry of Health, Community Development, Gender, Elderly, and Children (MoHCDEC)	1	03.0

*Sector*		
Health	16	48.5
Livestock	17	51.5

*Level of healthcare facility*		
District hospital	8	57.1
Regional hospital	5	35.7
District referral hospital (faith-based organisation)	1	07.1

## Data Availability

The data supporting the findings of this study are available from the corresponding author upon reasonable request.
